# *Agrobacterium*-mediated transformation systems of *Primula vulgaris*

**DOI:** 10.1186/s13007-018-0360-1

**Published:** 2018-10-26

**Authors:** Sadiye Hayta, Mark A. Smedley, Jinhong Li, Wendy A. Harwood, Philip M. Gilmartin

**Affiliations:** 1 School of Biological Sciences, University of East Anglia, Norwich Research Park, Norwich, NR4 7UH UK; 2 John Innes Centre, Norwich Research Park, Norwich, NR4 7UH UK; 3 The Earlham Institute, Norwich Research Park, Norwich, NR4 7UH UK

**Keywords:** *Primula vulgaris*, Heteromorphy, *Agrobacterium*, Transformation, Stable, Transient, Infiltration

## Abstract

**Background:**

Genetic transformation is a valuable tool and an important procedure in plant functional genomics contributing to gene discovery, allowing powerful insights into gene function and genetically controlled characteristics. *Primulaceae* species provide one of the best-known examples of heteromorphic flower development, a breeding system which has attracted considerable attention, including that of Charles Darwin. Molecular approaches, including plant transformation give the best opportunity to define and understand the role of genes involved in floral heteromorphy in the common primrose, *Primula vulgaris*, along with other *Primula* species.

**Results:**

Two transformation systems have been developed in *P. vulgaris*. The first system, *Agrobacterium*-mediated vacuum infiltration of seedlings, enables the rapid testing of transgenes, transiently *in planta*. GUS expression was observed in the cotyledons, true leaves, and roots of *Primula* seedlings. The second system is based on *Agrobacterium tumefaciens* infection of pedicel explants with an average transformation efficiency of 4.6%. This transformation system, based on regeneration and selection of transformants within in vitro culture, demonstrates stable transgene integration and transmission to the next generation.

**Conclusion:**

The two transformation systems reported here will aid fundamental research into important traits in *Primula*. Although, stable integration of transgenes is the ultimate goal for such analyses, transient gene expression via *Agrobacterium*-mediated DNA transfer, offers a simple and fast method to analyse transgene functions. The second system describes, for the first time, stable *Agrobacterium*-mediated transformation of *Primula vulgaris,* which will be key to characterising the genes responsible for the control of floral heteromorphy.

## Background

Genetic transformation is an important technique for the in vivo analysis of gene function, especially in relation to plant development through gene over-expression, gene knock-out, and gene structure function analyses. Additionally, the function of genes isolated using map-based cloning of mutant alleles has been confirmed by functional complementation using genetic transformation [1]. *Agrobacterium tumefaciens*-mediated transformation is a suitable technique to conduct such complementation analyses, offering defined integration of transgenes and single or low-copy number integration into transcriptionally active regions of the chromosome [2, 3]. Although stable integration of physiologically active and regulated transgenes is the ultimate goal of such functional studies, transient gene expression via *Agrobacterium*-mediated DNA transfer into different plant tissues offers an alternative route for the rapid analysis of transgene functions, which is also amenable to high-throughput analysis [4]. Transient systems like biolistic bombardment, protoplast fusion and *Agrobacterium*-mediated transient transformation each have advantages and disadvantages depending on the specific research requirements. Among these transformation assays tobacco leaf infiltration mediated by *A. tumefaciens* is widely appreciated due to its easy operation and high transformation efficiency [5].

Transgenic plants are usually produced by methods that include the transformation of individual plant cells followed by regeneration of whole plants from those transformed cells. This has the advantage of minimising the opportunity for chimeric transgenics, but the difficulty of plant regeneration from transformation-susceptible tissues is one of the key challenges in the production of transgenic plants [6]. *Primula* species are particularly recalcitrant to in vitro regeneration and this has limited the development of transformation systems in this genus. Until recently, only in vitro grown seedlings delivered explant materials that regenerated in tissue culture in various *Primula* species [7–11]. In *P. vulgaris,* in vitro regeneration was obtained only by destruction of the parent plant [12] and from pedicel explants [13]. Recently an efficient protocol was developed for in vitro regeneration of *P. vulgaris* via indirect organogenesis from adult leaf–derived explants without any seasonal limitations [14].

Following the recent identification of the *Primula S* locus that controls floral heteromorphy in *P. vulgaris* [15], the development of transformation systems for *P. vulgaris* is particularly important for the functional characterisation of the genes involved in the floral heteromorphy and self-incompatibility. Primulaceae species provide one of the best known examples of heteromorphic flower development; a breeding system has attracted considerable attention, including that of Charles Darwin [16, 17]. The common primrose, *P. vulgaris*, along with the majority of the approx. 450 species of *Primula* exhibit floral heteromorphy in which different individuals develop one of two possible forms of flower, known as pin and thrum. Both flower types are hermaphrodite and exhibit reciprocal positions of male and female reproductive structures, which together with an associated self-incompatibility system, promote outcrossing and inhibit self-pollination [18, 19].

Transgenic approaches give the best opportunity to define, understand and dissect the role of the genes involved in the control of floral heteromorphy in the common primrose, *P. vulgaris*, and other *Primula* species. The underlying genes responsible for these different flower forms control anther position, style length and pollen size, and are clustered within the *S* locus [20]. The recent isolation of the *S* locus supergene cluster, and its constituent genes [15], provides opportunities for their exploitation within crops following their functional characterisation. Potentially, transformation techniques for *Primula* species may provide opportunities for breeders to improve or create more horticultural varieties using molecular genetic knowledge.

*Primula* transformation is key to the important task of the functional definition of genes within the *S* locus [18]. In this paper, we describe two methods of genetic transformation for *P. vulgaris*. The first, vacuum infiltration of in vitro grown seedlings enables transient gene expression analysis. The second, transformation and in vitro regeneration of stably transformed *P. vulgaris* plants is key to unravelling the underlying mechanisms of *S* locus gene function.

## Results

**Fig. 1:**
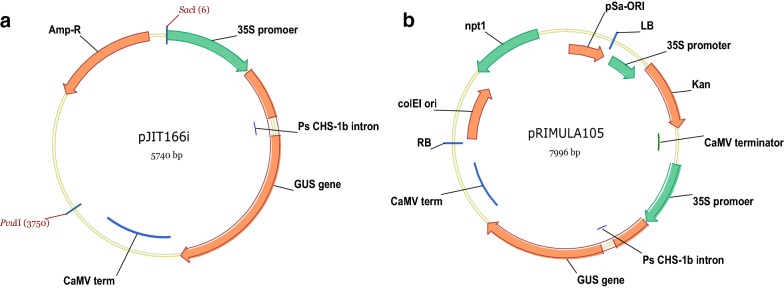
**a** The cloning vector pJIT166i containing the *CaMV 35S* promoter driving the *Uid*A (*GUS*) gene containing the *Pisum sativum CHS*-*1b* intron. **b** The *UidA* (*GUS*) reporter construct pRIMULA105 which was developed for the initiation and optimisation of *P. vulgaris* transformation

Our aim in this study was to establish a rapid transient system and reliable stable transformation system for *P. vulgaris*. To enable this, one of the first steps was to assess and build a reporter binary vector to be used for the optimisation of our transformation experiments (Fig. 1).

### Selection of CaMV *35S* promoter/*UidA* reporter gene cassette

**Fig. 2:**
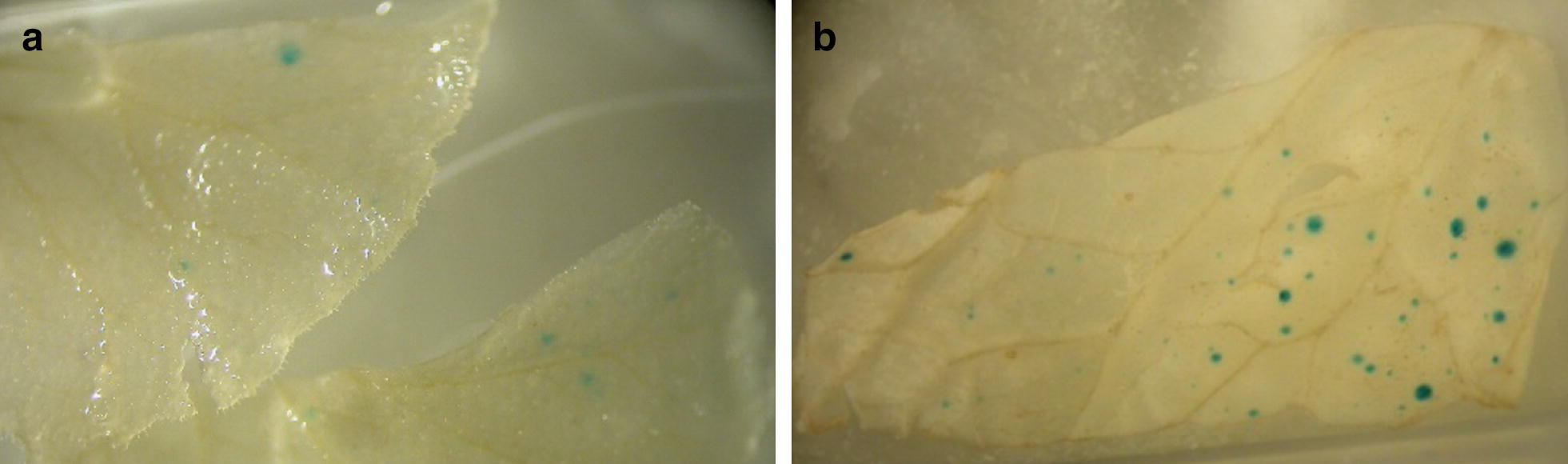
Transient GUS expression in *P. vulgaris* seedling leaves bombarded with: **a** pJIT161 containing the *Uid*A (*GUS*) gene without an intron, **b** pJIT161i containing the *UidA (GUS)* gene with the *Pisum sativum CHS*-*1b* intron

In initial biolistic transient expression experiments, we assessed different *35S*-*UidA* cassettes, and expression of the *UidA* reporter gene was visualised by a β-glucuronidase (GUS) assay. Details of the different constructs are provided in “Materials and methods” section. Blue foci could not be seen in the leaf sections bombarded with pBRACT104 [21] containing *35S*-*UidA* with the *Ricinus communis CAT1* intron. However, GUS foci were seen in the leaf material bombarded with pJIT161 [22] containing the *35S*-*UidA* gene without an intron (Fig. 2a). Material bombarded with pJIT166i containing the *Pisum sativum* CHS-*1b* intron produced more GUS foci and higher GUS accumulation was observed (Fig. 2b). These observations demonstrate that the CaMV *35S* promoter was active in *P. vulgaris*, that the *UidA* reporter gene was suitable for use in the optimisation of *Primula* transformation and that *P. vulgaris* processed the *CHS* intron1b within the *UidA* gene. The absence of GUS activity using *35S*-*UidA CAT1* intron cassette suggest that *P. vulgaris* cannot efficiently process the *R. communis CAT1* intron.

### *Agrobacterium tumefaciens* infiltration of *Primula vulgaris* seedlings as a transient assay system

**Fig. 3:**
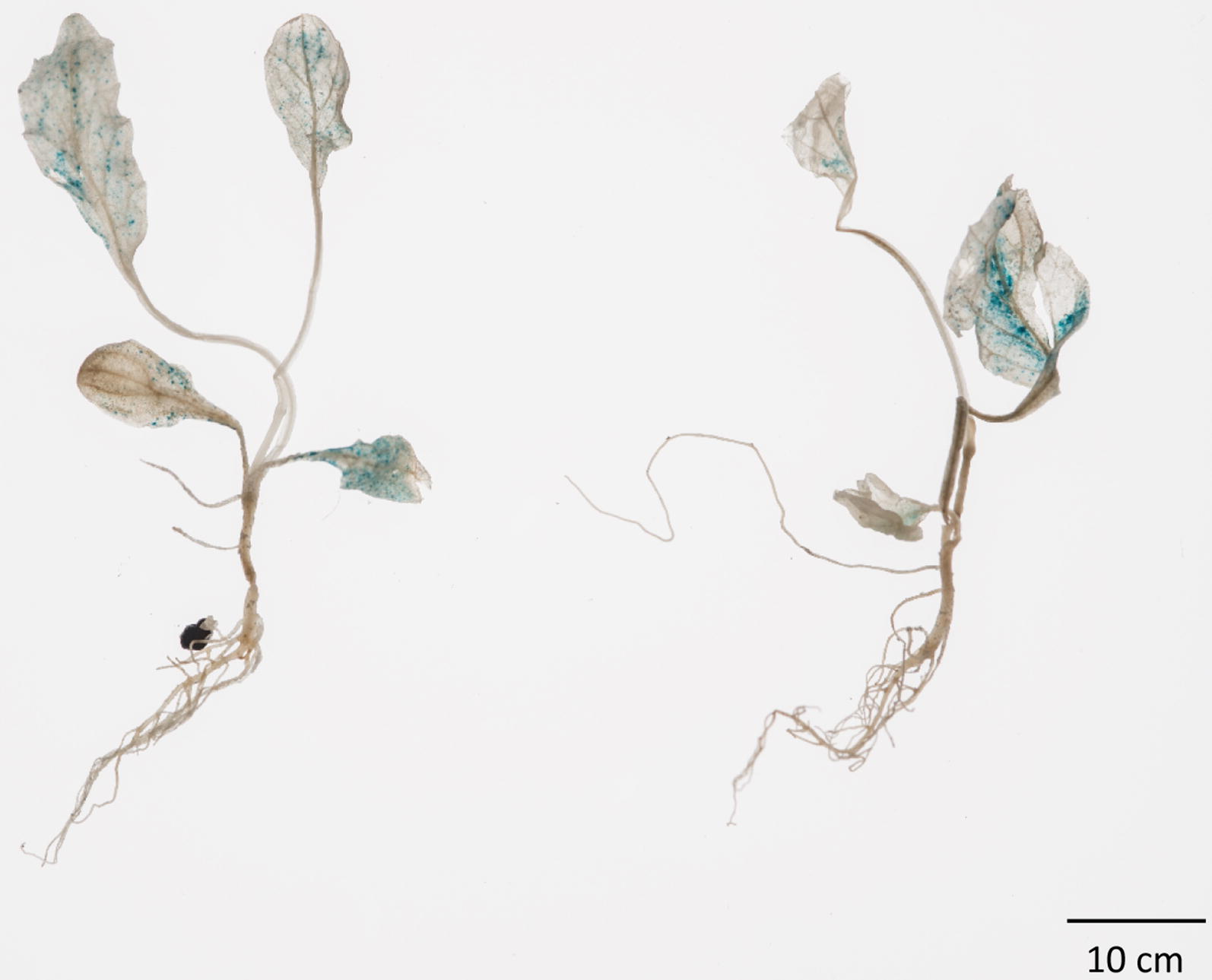
GUS expression in vacuum infiltrated seedlings. GUS assay of *P. vulgaris* seedlings 3 weeks after vacuum-infiltration with *Agrobacterium* AGL1 containing pRIMULA105

Having demonstrated functionality of the *35S*-*UidA CHS intron 1b* cassette, we cloned this into pBRACT102 to create a binary vector named pRIMULA105 for *Agrobacterium tumefaciens* mediated transformation assays in *P. vulgaris*. GUS activity was observed in the cotyledons, true leaves, and some root parts of *P. vulgaris* seedlings after infiltration with *A. tumefaciens* AGL1 containing pRIMULA105 and in vitro cultured on solid MS medium (Fig. 3). Optimal GUS activity was seen in seedlings infiltrated at the two-true leaf stage, where the largest of the true leaves had reached approximately 0.5 cm^2^ (15 seedlings out of 15). A considerable decrease or no GUS activity was seen in seedlings infiltrated after the four-true leaf stage or later (2 seedlings out of 15).

### Stable *Agrobacterium tumefaciens*- mediated *Primula vulgaris* transformation

#### Selection of best explant for stable transformation systems

Leaf material as described in Hayta et al. [14] and pedicel explants were compared in our first experiments. When the leaf-derived explant was used, sensitivity to *Agrobacterium* was observed and the elimination of *Agrobacterium* proved difficult when compared to pedicel derived material (data not shown). Therefore, subsequent work concentrated on the use of pedicel explants.

### Explant pre-culture prior to inoculation with *Agrobacterium tumefaciens*

Pre-culturing explants for 2 weeks prior to inoculation with *A. tumefaciens* was found to be crucial for explant viability and subsequent shoot regeneration. Explants inoculated with *A. tumefaciens* on the day of isolation often resulted in a hypersensitive response showing browning and a loss of viability, ultimately resulting in explant death. Overgrowth of these explants by *A. tumefaciens* was common and latent bacterial contamination was also observed. Explants pre-cultured for 2 weeks prior to *A. tumefaciens* inoculation began to swell and callus prior to inoculation. Explants suffering with latent bacterial contamination were identified after 2 weeks pre-culture and discarded. Pre-cultured explants showed reduced overgrowth from *A. tumefaciens* and less sensitivity to the bacterial treatment. The viability of the explant receiving the pre-culture treatment was improved. GUS activity on pre-treated pedicel explants was 68% after 4 weeks, this was reduced to 3.3% on non-pre-cultured explants mainly because of *A. tumefaciens* overgrowth.

### Optimisation of selection conditions for *P. vulgaris*

Sensitivity to the selective agent kanamycin was determined by including different concentrations of kanamycin (0, 100, 125, 150, 200 mg L^−1^) in PCI medium and visually assessing plant material after 3 weeks culture. Kanamycin at 200 mg L^−1^ completely inhibited callus growth and caused severe necrosis. On PCI medium lacking kanamycin, 100% callus induction was obtained from the cultured explants. The inclusion of 100 mg L^−1^ kanamycin moderately checked callus growth. Inclusion of 150 mg L^−1^ kanamycin inhibited callus growth and cause minor necrosis. Kanamycin at a concentration of 125 mg L^−1^ checked callus growth but did not cause necrosis. Therefore, a concentration of 125 mg L^−1^ kanamycin was deemed to be a suitable level of selection for subsequent transformation experiments.

### Inclusion of AgNO_3_ in co-cultivation medium

**Fig. 4:**
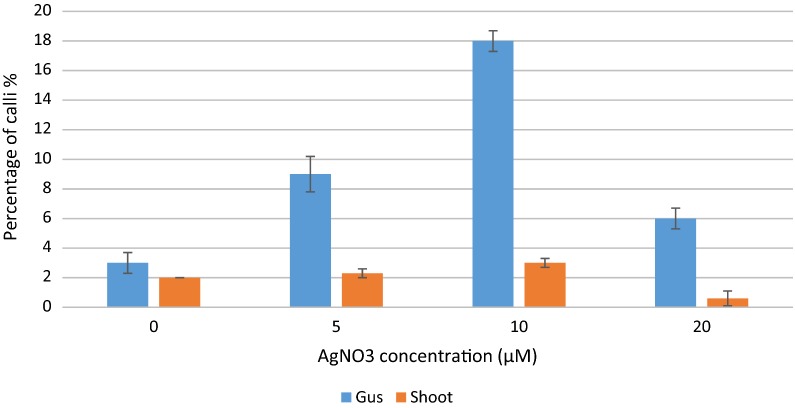
Effect of different concentrations of AgNO_3_, included in co-cultivation medium, on percentage of calli showing GUS expression (blue) and shoot regeneration (orange) of *Primula vulgaris*. Percentage of calli producing shoots from three replicates with a total of 45 calli per treatment (n = 45). Error bars show the SD from mean values of shoot production and GUS expression

**Fig. 5:**
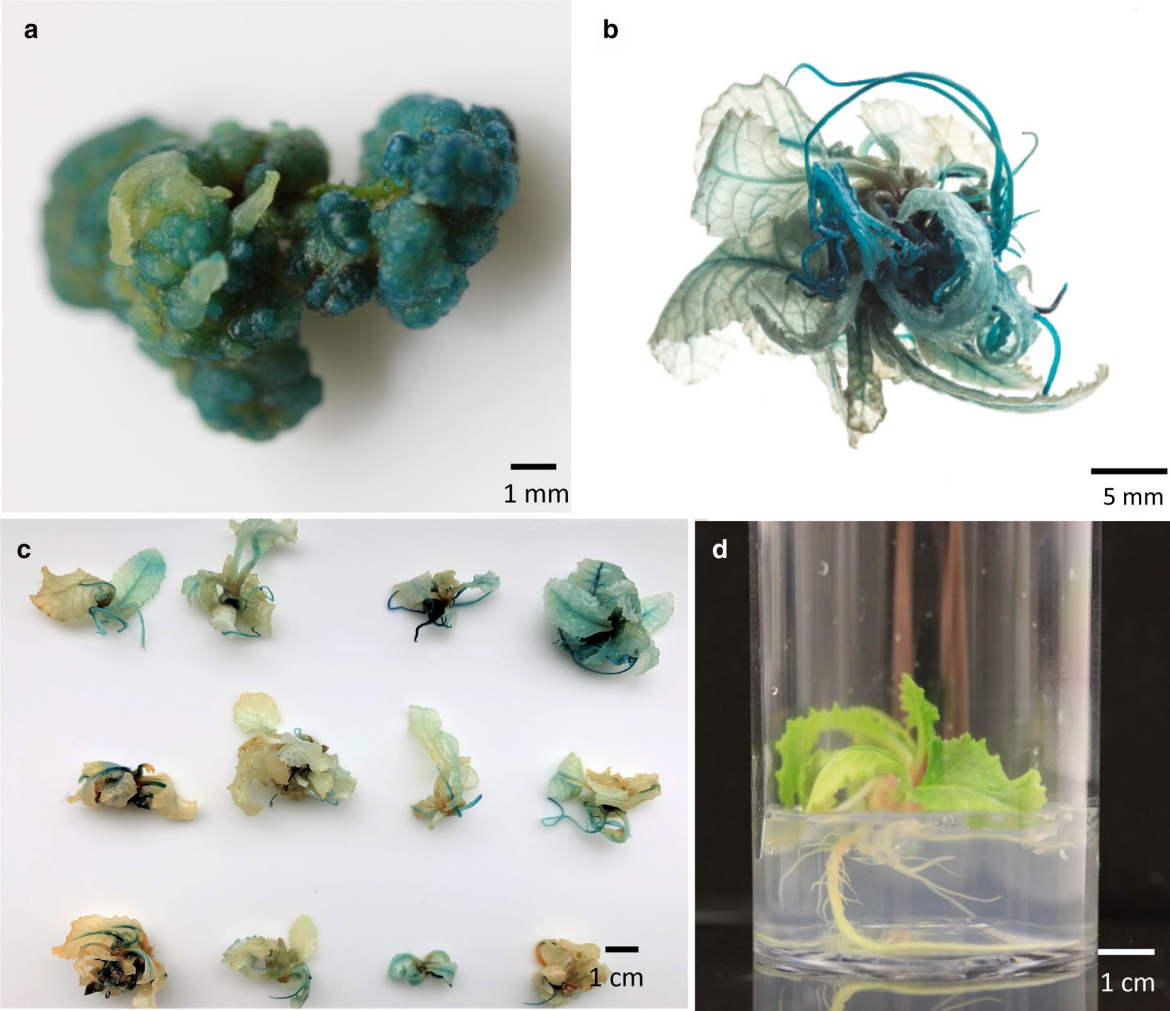
GUS expression in stable transformed lines. **a** GUS expression in sectors of compact callus—10 µM AgNO_3_ was included in the co-cultivation medium. **b** Regenerated transgenic plantlet showing strong GUS expression. **c** Twelve independent transgenic lines showing GUS expression. **d** Transgenic plantlet rooting in WPM with 135 mg L^−1^ kanamycin. Plantlet shows strong root formation with multiple root hairs on the selection medium

The inclusion of AgNO_3_ in co-cultivation medium had a dramatic effect on shoot regeneration and the percentage of explants showing GUS expression. A reduction of explants overgrown by *A. tumefaciens* was also observed. AgNO_3_ was included at concentrations of 5, 10 and 20 µM in co-cultivation medium. Calli showing shoot regeneration increased with the addition of AgNO_3_ to co-cultivation medium, except at the higher concentration of 20 µM (Fig. 4). The percentage of explants showing GUS activity increased when AgNO_3_ was included in co-cultivation medium compared to medium without AgNO_3_ (Fig. 4). Inclusion of AgNO_3_ at a concentration of 10 µM had the greatest effect on shoot regeneration and calli showing GUS activity compared to all other treatments, 3% and 18% respectively. Strong GUS activity was seen in the compact calli stained after 8 weeks in vitro culture (Fig. 5a). Supplementation of PCI media with additional copper sulphate (CuSO_4_) had no effect on shoot regeneration, whether combined with or without AgNO_3_ (data not shown).

### Transformation efficiencies

With a combination of 2 weeks’ pre-culture, inclusion of AgNO_3_ at 10 µM in the co-cultivation medium and selection set at 125 mg L^−1^ kanamycin during callus induction and shoot regeneration; 37.8% of pedicel explants developed green organogenic calli. Three hundred and two pedicel explants inoculated with *A. tumefaciens* containing pRIMULA105 produced 55 rooted putative transformants on WPM with 125 mg L^−1^ kanamycin. PCR analysis identified 14 of these to contain the *NptII* gene, giving an overall transformation efficiency of 4.6%. All samples were shown to be free of *A. tumefaciens* contamination by *VirD2* PCR analysis. A positive *P. vulgaris Actin* gene PCR control proved all DNA extractions were of PCR amplifiable quality (data not shown). A subset of the *NptII* PCR positive plants were GUS stained and showed strong GUS activity (5b). Twelve independent transgenic lines displaying GUS expression are shown in Fig. 5c. Subsequently, increasing the kanamycin level to 135 mg L^−1^ in rooting medium (Fig. 5d) proved effective in reducing the number of escapes without affecting the overall transformation efficiency. It was observed that non-transformed plantlets (escapes) did not produce root hairs when grown on rooting medium with kanamycin selection, whereas transformed plantlets displayed strong roots with an abundance of root hair (confirmed by PCR analysis).

### Analysis of transgene inheritance

To test that the inherited transgene’s expression was stable in the subsequent generation, T_1_ seed from five outcrossed T_0_ lines were germinated on kanamycin containing MS medium. Kanamycin resistant seedlings were obtained from each of the five lines, demonstrating stable transgene expression in the next generation (data not shown). To further address stable inheritance of the transgene, we used Southern analysis of the progeny of one of these transgenic lines. As described above, *P. vulgaris* plants produce either pin or thrum form flowers. A thrum background transgenic T_0_ plant was outcrossed to a pin background non-transgenic plant to produce T_1_ seeds and the progeny grown in order to confirm inheritance of the transgene in the next generation. Transgene inheritance in the T_1_ progeny was confirmed by *NptII* PCR analysis. Samples were determined to be free of *A. tumefaciens* contamination by the lack of an amplification product from the *VirD2* PCR, alongside control PCR amplifications using the *P. vulgaris Actin* gene. Of the 54 T_1_ plants tested, PCR analysis confirmed that 28 of them, approximately 50%, had inherited the *NptII* selection gene. This demonstrates stable transgene transmission to the next generation with a Mendelian inheritance ratio of 1–1 and suggests transgene insertion at a single locus.

### Southern blot hybridization

**Fig. 6:**
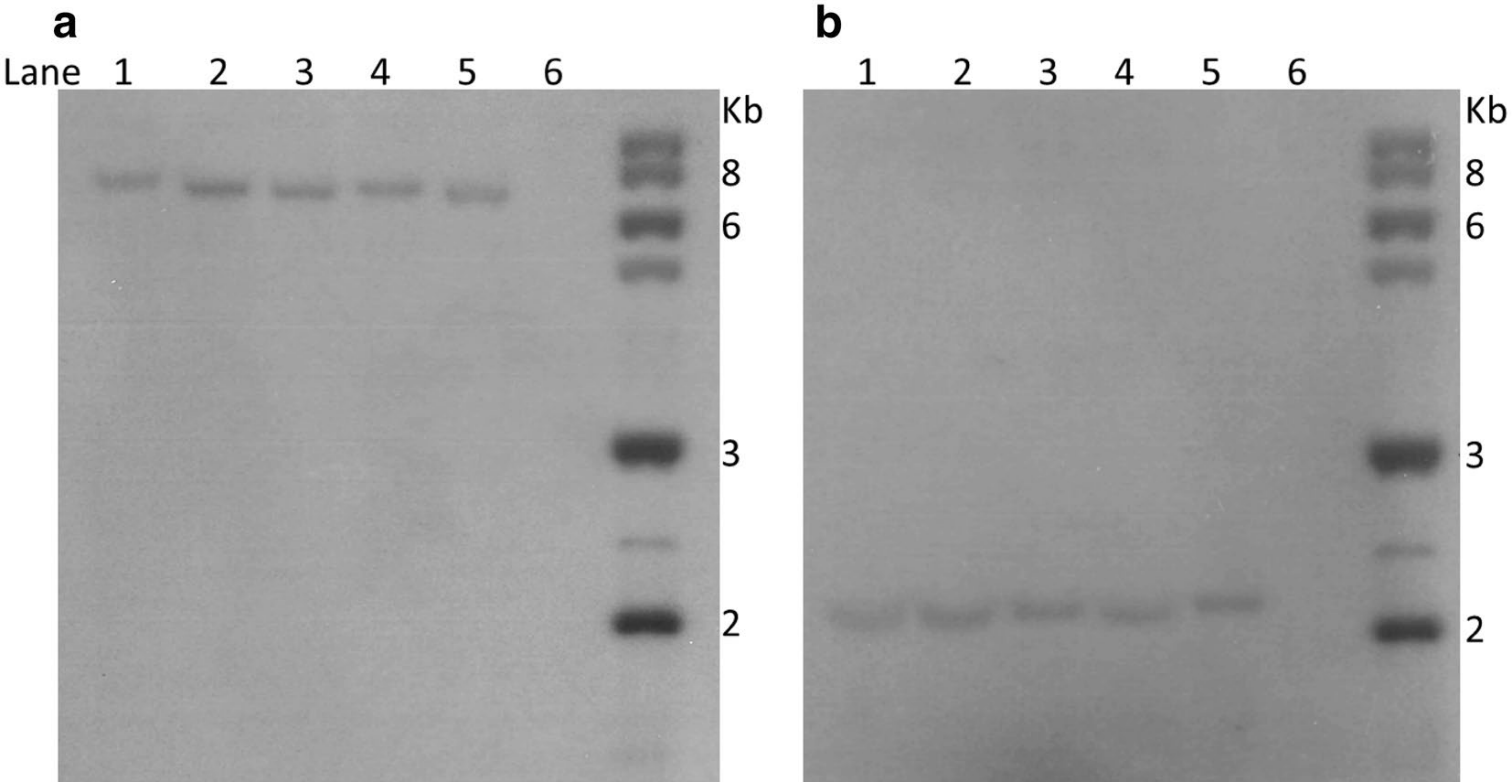
Southern analysis, using nptII probe, showing transgene inheritance. Genomic DNA digested with restriction enzyme **a** *Hind*III and **b** *Eco*RI. Lanes 1–4, T1 generation transgenic plants, Lane 5, parental T0 primary transgenic plant. Lane 6, WT control

To further confirm the transformation of the T_0_ line and the T_1_ progeny, southern blot analysis was performed on a subset of four *NptII* PCR positive progeny plants (Fig. 6). A single transgene insertion was observed in the T_0_ plant and its four T_1_ progeny reaffirming the previous PCR segregation data. No hybridisation signal was observed in the non-transformed *P. vulgaris* negative control. These results confirm the integration of the *NptII* gene into the *P. vulgaris* genome at a single locus and prove transgene inheritance to the next generation.

## Discussion

We have developed two transformation systems for *P. vulgaris*. The first method involves the infiltration of *Primula* seedlings for transient gene expression studies. The use of infiltration of seedlings by *A. tumefaciens* was described as a simple transient expression in *Arabidopsis thaliana* [5], and called FAST “Fast *Agro*-mediated Seedling Transformation”. There are only a few species for which FAST methods are available. In the model plant *A. thaliana* seedling infiltration has been used to express a wide variety of genes driven by many different promoters in *A. thaliana* seedling cotyledons [5]. For the model legume *Medicago truncatula,* seedling infiltration was developed and has provided a wide range of opportunities for genetic analysis [6]. A recent adaptation of the FAST method was used in transient overexpression experiments in *Catharanthus roseus* in order to study secondary metabolism [23]. Transient gene silencing was performed by injecting *P. forbesii* with a virus induced gene silencing (VIGS) vector [24], however, this methodology has limited scope when compared to the opportunities offered by FAST, such as over expression, promoter testing and RNAi [5]. Transient gene expression systems used in functional genetic assays allow rapid gene characterization with lower inputs than creating stable transformed plants, especially if the plants have long generation cycles like *P. vulgaris* [25, 26]. This transient expression system, when compared with a stable tissue-culture-based stable transformation method, is technically easier and faster, as it does not require tissue culture-based regeneration procedures, although it does not routinely lead to germline transformation which is important for some studies. The second method describes, for the first time, stable *A. tumefaciens*-mediated transformation of *P. vulgaris* and enables stable transgene integration and transmission to the next generation. Transgene transmission followed a Mendelian inheritance ratio of 1:1 as expected in an obligate outcrossing species such as *P. vulgaris*. Stable expression of the *Npt*II transgene was observed in the next generation as assessed by kanamycin resistance in T_1_ seedlings.

Primulaceae species provide the best-known examples of heteromorphic flower development, and as such, offer the opportunity to be able to define and understand the role of genes involved in floral heteromorphy [15, 27]. *P. vulgaris* plants either produce pin form flowers or thrum form flowers and crosses only occur between these two different forms. The underlying genes responsible for these different flower forms control anther position, style length and pollen size, and are clustered within the *S* locus [15]. Improvements in large scale DNA sequencing has expedited the molecular characterisation of floral heteromorphy in *Primula* and other species [15, 24, 28]. Recently, the *Primula S* locus was shown to be a supergene comprising of a tightly linked cluster of five thrum-specific genes, spanning a 278-kb sequence that is absent in plants producing the pin flower form [15]. *P. vulgaris* transformation will prove the key to ascribing gene functionality to the different genes within the *S* locus supergene,

Many factors may affect the genetic transformation efficiency of plants. One crucial factor is the regeneration potential of the target material [6]. *Primula* species are particularly recalcitrant to in vitro regeneration and this has limited the development of transformation systems in *Primula* species. In *P. vulgaris* in vitro regeneration was obtained only by destruction of the parent plant [12] and from pedicel explants [13]. An efficient protocol was developed for in vitro regeneration of *P. vulgaris* via indirect organogenesis from adult leaf–derived explants without seasonal limitations [14]. Although, leaf-derived explants gave efficient regeneration, they were found to not be suitable for stable transformation because of their sensitivity to bacterial treatment and overgrowth from *A. tumefaciens*. To date, there are no published stable transformation studies in species of the *Primulaceae*.

Another important factor affecting transformation efficiency is the use of an effective selective agent that inhibits the development of non-transformed cells and permits the growth of transformed tissues. The selective agent concentration in culture medium can influence shoot regeneration and high concentrations may promote adverse effects on the organogenic potential of plant material. The most common selectable marker genes used in plant transformation vectors include constructs providing resistance to antibiotics such as kanamycin and hygromycin B and the *Bar* gene that allows the growth of plant cells in the presence of herbicides such as phosphinothricin (PPT), bialaphos or similar compounds.

The *neomycin phosphotransferase II* (*NptII*) gene has proven to be the most widely applicable and has been used in most transformation procedures for dicotyledonous plants. The *NptII* gene confers resistance to aminoglycoside antibiotics kanamycin, neomycin and G-418, of which, kanamycin is the most used selective agent. Of the three major selection systems, the *NptII* gene and kanamycin selection seemed the most appropriate to initially use with *P. vulgaris*. Kanamycin at a level of 125 mg L^−1^ inhibited the growth of non-transformed cells while enabling transformed cells to regenerate from pedicel callus explants. As explants regenerated and grew they were moved onto rooting medium with level of kanamycin to 135 mg L^−1^. Maintaining explants for some additional 2–3 weeks on this selective rooting medium reduced the average number of escapes to around 12% of putative transformants. It was also observed that transformed plantlets, on kanamycin selection, could easily be identified early during the rooting process by their production of root hairs, which was later confirmed by PCR analysis.

Pre-culture of explants was found crucial in this study, improving explant viability, reducing explant overgrowth from *A. tumefaciens* and assisting in the identification and removal of bacterial contaminated material prior to *A. tumefaciens* inoculation. Pre-induction of the regeneration capacity via a pre-culture treatment has been reported to improve the adhesion of *A. tumefaciens* during co-cultivation [29]. Pre-culture activates cell division and the phase of the cell-cycle has been shown to influence stable transformation [30] The presence of actively dividing cells improved cell competence for *A. tumefaciens* infection in regenerable tissue [29]. In *Brassica rapa*, seedling hypocotyls pre-cultured prior to exposure to *A. tumefaciens* showed greater shoot regeneration and improved transformation efficiency [31]. Similar improvements in regeneration and transformation efficiencies were reported for pre-cultured explants in tomato *Solanum lycopersicum* [32] and potato *S. tuberosum* [33].

The availability of good quality donor material for the initiation of explants was essential for maintaining *P. vulgaris* transformation efficiencies. Poor donor material or parent plants that had had a fungicidal application led to poor transformation efficiency in experiments (data not shown).

The addition of AgNO_3_ in co-cultivation medium improved transformation efficiency, helped to prevent *A. tumefaciens* overgrowth, and reduced browning of the explant material. The addition of AgNO_3_ was found effective in combination with Timentin for the control of *A. tumefaciens* in selection medium. Potent ethylene inhibitors, such as AgNO_3_, are added to plant media for enhancing shoot regeneration and preventing the negative effects of the gaseous plant hormone ethylene, extensively reviewed by Kumar et al. [34]. In addition, AgNO_3_ has been reported to promote callus proliferation [35], and improve root formation [36], and is widely used within plant tissue culture to aid plant regeneration in both dicot and monocot in vitro cultures [34]. In other species, AgNO_3_ inhibited shoot regeneration [37] which would indicate that the promotive function of AgNO_3_ on shoot regeneration is species-specific [38]. The addition of AgNO_3_ remarkably enhanced regeneration of *P. vulgaris* within tissue culture [14]. Silver ions play a major role in influencing somatic embryogenesis, shoot formation, and efficient root formation, all of which are prerequisites for successful plant transformation [34]. In addition, silver ions are effective inhibitors of microbial growth [39], AgNO_3_ was found to inhibit *A. tumefaciens* growth due to its bactericidal properties [40]. Effective control and or elimination of *A. tumefaciens* after co-cultivation was reported when AgNO_3_ was used in combination with antibiotics [40]. Mendoza-De Gyves et al. [40] concluded that the advantages of adding AgNO_3_ within plant transformation systems were the inhibition of *A. tumefaciens* growth and the stimulation of plant regeneration.

## Conclusions

In summary, we have developed two transformation systems for *P. vulgaris*, that will aid fundamental research into important traits in this species. The first system, vacuum infiltration of seedlings, enables the rapid testing of transgenes, *in planta*. This transient gene expression protocol is a useful tool for testing gene function and for reporter gene expression. The second transformation system allows the stable transformation of *P. vulgaris* using pedicel explants as its starting material and offers, for the first time, the methodology to allow the identification, and functional characterisation, of genes that are involved in the control of floral heteromorphy within *Primula* species.

## Materials and methods

### Vectors and *Agrobacterium* strain

Three vectors all containing the *35S* promoter [41] driving expression of the *UidA* (*GUS*) [42] gene; pJIT166 [22] containing the 2×*35S* promoter driving the original *Uid*A (*GUS*) gene without an intron, pJIT166i containing the 2×*35S* promoter driving the *UidA* (*GUS*) plus the *Pisum sativum Chalcone Synthase* (*CHS*) intron1b (Fig. 1a) and pBRACT104 [21] containing the *35S* promoter driving the *UidA* (*GUS*) gene plus the *Ricinus communis Catalase* intron 1 (*CAT1*) were assessed, for use in *Primula*, by biolistic bombardment.

The pBRACT100 series of vectors [21], containing the *Neomycin Phosphotransferase II* (*NptII*) gene, which confers *in planta* resistance to kanamycin, driven by the CaMV *35S* promoter (*35S*) were used for these studies. The pBRACT102 vector, containing the 35S::*NptII* and a Multiple Cloning Site (MSC), was modified to produce a reporter construct for use in *Primula*. The 2×*35S* promoter, β-glucuronidase (*UidA/GUS*) coding region containing the Pea (*Pisum sativum*) *Chalcone Synthase* (*CHS*) *1b* intron and the CaMV terminator were isolated as one 3744 bp fragment from pJIT166i using *Sac* I and *Pvu* II. The fragment (2×*35S*::*GUS* intron::CaMV terminator) was subsequently cloned into the *Sac* I and *Stu* I sites of pBRACT102. The resulting plasmid was named pRIMULA105 (Fig. 1b).

The hypervirulent *Agrobacterium tumefaciens* strain AGL1 [43] containing pRIMULA105 was used to establish and optimise the plant transformation experiments. The vector pRIMULA105 was electroporated into *A. tumefaciens* AGL1 cells as described by Bartlett et al. [44] along with the helper plasmid pSoup [45]. A modified method of [46] was used to prepare standard *A. tumefaciens* inoculums for transformation as previously described by Bartlett et al. [44]. Equal quantities of the *A. tumefaciens* culture and 30% sterile glycerol were mixed and made into 200 μl aliquots in 0.5 mL Eppendorf tubes. The standard inoculum aliquots were frozen by placing at − 80 °C and stored.

### Particle bombardment for construct testing

Particle bombardment was used to test the constitutive CaMV *35S* promoter and the *UidA* (*GUS*) reporter gene containing different introns in *P. vulgaris*. Young leaf material was excised from adult plants under aseptic conditions and surface sterilised as described by Hayta et al. [14]. The leaf material was then cut into approximately 1.6 cm^2^ sections and placed centrally onto solidified MS medium in 9 cm diameter Petri dishes. Three constructs were assessed pJIT166, pJIT166i and pBRACT104. Approximately 1 µg of plasmid DNA was coated onto gold particles and bombarded into the leaf material using the method and parameters as described in Harwood and Smedley [47]. Each leaf section was bombarded twice, being turned 180 degrees between bombardments, three leaf sections were bombarded with each plasmid. The negative control consisted of leaf sections bombarded with gold particles without DNA. The petri dishes were sealed, and the leaf sections cultured for 3 days at 21 ± 1 °C under cool white fluorescent light (50 μmol m^−2^ s^−1^) with a 16-h light period per day prior to assessment by GUS assay.

### Histochemical GUS staining

Histochemical staining of GUS activity was performed by incubating plant tissue at 37 °C overnight (~ 16 h) in 1.0 mg mL^−1^ 5-bromo-4-chloro-3 indolyl-D-glucuronic acid, 0.1 M Na_2_HPO_4_ buffer (pH = 7.0), 0.5 mM K_3_(Fe(CN)_6_), 0.5 mM K_4_(Fe(CN)_6_), and 10 mM EDTA as described by Jefferson et al. [48]. The chlorophyll was removed from the plant tissues by immersing in 100% ethanol and incubating at room temperature 24 ± 1 °C with gentle agitation for approximately 6 h. The ethanol was changed twice during incubation. The GUS-positive plant tissues were examined with a bright field microscope (Leica MZ6, Cambridge, England) at a low magnification and photographed with a digital camera (Nikon Coolpix 4500, Japan).

### Transient expression system

#### Plant material and explant preparation

Under aseptic conditions, 0.5 g of *P. vulgaris* seeds were surface sterilized in a 1.5 mL Eppendorf tube with 70% (v/v) ethanol for 2 min and rinsed with sterile-distilled water, incubated for 20 min in 10% (v/v) sodium hypochlorite (Fluka 71696) with a drop of Tween 20 (Sigma P‐9416) with gently agitation. The seed were subsequently rinsed three times with distilled-sterile water. To aid seed germination, the seeds were pre-treated by applying filter sterilised gibberellic acid (GA_3_) at 400 ppm and incubated at room temperature 24 ± 1 °C for 16 h in the dark. The GA_3_ solution was removed and discarded. Seeds were transferred to 100 mL Sterilin jars (5 per jar) containing 20 mL Seed Germination (SG) medium (described below). Seed germination took place in a controlled culture room at 21 ± 1 °C under cool white fluorescent light (50 μmol m^−2^ s^−1^) with a 16-h light period per day. Seedlings were cultured for approximately 4 weeks prior to infiltration.

#### Media

Seed Germination (SG) medium, Murashige and Skoog (MS) [49] basal medium containing 3% (w/v) sucrose and solidified with 0.6% (w/v) agar, without plant growth regulators.

#### Preparation of *Agrobacterium tumefaciens*

A 200 μL aliquot of standard inoculum was removed from − 80 °C storage, added to 10 mL of LB liquid medium containing 25 μg mL^−1^ kanamycin and 25 μg mL^−1^ rifampicin and incubated at 28 °C shaken at 200 rpm for 24 h. Five hundred microliters of this culture were used to inoculate 50 mL of LB liquid medium supplemented with 25 μg mL^−1^ kanamycin, 25 μg mL^−1^ rifampicin and 5 µM acetosyringone (AS) incubated at 28 °C and shaken 200 rpm overnight ~ 16 h. The *A. tumefaciens* culture was transferred to 50 mL Falcon tubes (25 mL^−1^ tube) and centrifuged at 3100 rpm for 10 min at 24 °C, to pellet the bacteria. The supernatant was discarded and the bacterial pellets were resuspended in 10 mM MgCl_2_ pH 5.8 containing 100 µM AS. The optical density of the infiltration buffer containing *Agrobacterium* cells was adjusted to 0.5 at OD_600_. Each 50 mL Falcon tube contained 25 mL infiltration buffer and the resuspended *A. tumefaciens* cells. The Falcon tubes were placed horizontally on a shaker and gently shaken at 120 rpm at room temperature 24 ± 1 °C for 4 to 5 h. Immediately prior to seedling infiltration Silweet ^®^L-77 was added to the *A. tumefaciens* cells to a final concentration of 0.01%.

#### Vacuum infiltration of *Primula vulgaris* seedlings

Under aseptic conditions, between 5 and 7 in vitro grown seedlings were placed in a 50 mL Falcon tube containing 25 mL of *A. tumefaciens* suspension containing pRIMULA105 prepared in infiltration buffer. The open Falcon tube, in turn, was placed within a sterile desiccator attached to a vacuum pump within a laminar air-flow cabinet. A vacuum was drawn to 600 mm Hg and held for 15 min, the vacuum was released rapidly, and the procedure repeated four times over 1 h. Following the four exposures to vacuum, the tube was left to sit at ambient air pressure for 20 min. The bacterial suspension was discarded, and the seedlings were washed six times with sterile-distilled water to remove excess *A. tumefaciens.* The seedlings were transferred to a sterile 1 L Erlenmeyer flask containing 250 mL of liquid MS medium with 3% sucrose and incubated at room temperature with gentle agitation (60 rpm). After 24 h, filter sterilised cefotaxime was added to the MS medium at a final concentration of 250 mg L^−1^ to control *A. tumefaciens* growth. The seedlings were maintained in the liquid MS at room temperature with gentle agitation (60 rpm) for a further 2 days (48 h). The seedlings were then assayed for GUS expression as described previously.

### Stable transformation of *Primula vulgaris*

#### Plant material and explant preparation

Adult flowering plants of *P. vulgaris* were used as the initial explant source for this study. Parent plants were grown in 1 litre pots of peat-based multi-purpose compost (Erin Horticulture, Ireland—MPC60) in a glasshouse at 16 ± 1 °C day and night, relative humidity (RH) 60% under natural light. Pedicels were harvested when the flower buds were closed and just showing the tips of the petals (~ 1–2 mm of yellow showing). Under aseptic conditions within a laminar flow cabinet, Pedicels with the buds attached, were surface sterilized by immersion in 70% (v/v) ethanol for 1 min and rinsed once with sterile-distilled water. This procedure was followed by immersion in 10% (v/v) bleach (10% sodium hypochlorite; Fluka 71696) with a drop of Tween 20 (Sigma P‐9416), for 12 min, followed by six rinses with distilled-sterile water. Tissue damaged by the bleach was excised and discarded before cutting each pedicel into 0.3–0.5 cm sectional explants, the flower buds were discarded. Explants were placed horizontally on PCI medium as a pre-cultivation treatment before inoculation. Twenty explants were cultured per 9 cm Petri plate. These samples were cultured under low light conditions (12.1 μmol m^−2^ s^−1^) at 21 ± 1 °C with a 16-h photoperiod. The explants were cultured for 2 weeks on PCI medium prior to inoculation with *A. tumefaciens* containing the relevant Ti plasmid.

#### Media

All media were prepared at double (2X) the final required concentration, adjusted to pH 5.8 and filter sterilized. Phytagel (catalog no. P8169; Sigma-Aldrich Co., St. Louis, MO, USA), used as the gelling agent, was also prepared at double final required concentration, autoclaved, and stored at room temperature until required. To prepare the final medium, both double-concentration Phytagel and media components were heated to 60 °C in a water bath, then mixed together, and poured into 9 cm diameter petri dishes or Sterilin jars.

Primula Callus Induction (PCI) medium [14], consisted of Gamborg (B5) [50] macro salts with 965.77 g L^−1^ KNO_3_, ½ MS micro salts and MS vitamins, 20 µM AgNO_3_, 3 mg L^−1^ thidizuron (TDZ), 0.3 mg L^−1^ α-naphthaleneacetic acid (NAA), 30 g L^−1^ Maltose, 3.5 g L^−1^ phytagel.

Primula rooting medium (PRM) [14], consisted of woody plant medium (WPM) (Lloyd and McCown 1981) basal salts with 2% sucrose, 0.5 mg L^−1^ indole-3-butyric acid (IBA), solidified with 1.75 g L^−1^ Phytagel.

#### Preparation of *Agrobacterium tumefaciens*

One hundred microliters of standard inoculum was transferred to 10 mL of MG/L [51] (tryptone, 2.5 g L^−1^ yeast, 100 mg L^−1^ NaCl, 5 g L^−1^ mannitol, 1 g L^−1^ Glutamic acid, 250 mg L^−1^ KH_2_PO_4_, 100 mg L^−1^ MgSO_4_, 1 μg L^−1^ Biotin. pH = 7) liquid medium without antibiotics. This was grown overnight ~ 16 h at 28 °C shaken at 200 rpm. The bacterial optical density was adjusted to 1.0 at OD_600_ using sterile MG/L prior to explant inoculation.

#### Determination of sensitivity of *Primula vulgaris* to the selective agent kanamycin

Pedicel explants that had begun to swell and cells divide following precultured on PCI medium for 4 weeks were used to assess the sensitivity of *P. vulgaris* to the selective agent kanamycin. Kanamycin was included into the PCI medium at different concentrations: 0, 100, 125, 150, 200 mg L^−1^. Fifteen precultured explants were used per 9 cm Petri plate with 3 petri plates per treatment and cultured at 21 ± 1 °C under low light conditions (12.1 μmol m^−2^ s^−1^) with a 16-h photoperiod. The material was subcultured, after 2 weeks, onto fresh PCI with the different kanamycin concentrations. The plant material was visually assessed and treatments compared after 3 weeks of culture.

#### Explant inoculation with *Agrobacterium tumefaciens*

Under aseptic conditions the pre-cultured pedicel explants were immersed fully in the MG/L-*A. tumefaciens* suspension and incubated at room temperature for 5 min. The explants were blot dried on sterile filter paper to remove excess *A. tumefaciens* before transferring, 15 per plate, to co-cultivation medium consisting of PCI supplemented with 200 μM AS and 10 µM AgNO_3_. The plates were co-cultured in the dark at 23 ± 1 °C for 3 days. After co-cultivation, the explants were transferred to PCI medium containing 160 mg L^−1^ Timentin for elimination of *A. tumefaciens* and 20 µM AgNO_3_ for a 5 day resting period. The cultures were then incubated at 21 ± 1 °C under cool white florescent light (100 mmol m^−2^ s^−1^) with a 16-h photoperiod.

#### Selection and plantlet regeneration

After a 5-day resting period, the explants were transferred onto selection medium consisting of PCI medium containing 20 µM AgNO_3_, Timentin (160 mg L^−1^) and kanamycin (125 mg L^−1^). The explants were subcultured onto fresh medium fortnightly. After 4–6 weeks the callus explants began to produce adventitious shoots. Shoots longer than 10 mm were isolated and transferred individually to 100 mL Sterilin jars containing 20 mL of PRM supplemented with 25 mg L^−1^ kanamycin and 160 mg L^−1^ Timentin. Plantlets were maintained for 4–6 weeks in Sterilin jars, incubated at 21 ± 1 °C under cool white florescent light (100 mmol m^−2^ s^−1^) with a 16-h photoperiod.

#### Transfer and establishment in soil

Well-rooted, healthy looking plantlets were transferred to multi-purpose compost in 5 cm square cells within propagation trays. Plants were covered with clear plastic propagator lids for approximately 2 weeks to maintain high humidity around them while they established in soil. Plants were grown in a controlled environment room at 18 ± 1 °C day and 15 ± 1 °C night temperatures, 75% relative humidity with light intensity of 250 μmol m^−2^ s^−1^ provided by metal halide lamps (HQI) supplemented with tungsten bulbs with a 16 h photoperiod.

#### DNA extraction

Leaf samples with a mass of approximately 100 mg were harvested into 1.5 mL Eppendorf tubes and flash frozen using liquid nitrogen. If DNA extraction could not be carried out immediately then the leaf material was stored at − 80 °C. The DNA was extracted from the leaf material using the Qiagen DNeasy plant mini kit (Cat No. 69106) following the manufacturer’s instructions. The DNA concentrations were assessed using the Nanodrop ND-1000 spectrophotometer.

#### Polymerase chain reaction (PCR)

Polymerase chain reactions were prepared using RedTaq ReadyMix PCR Reaction Mix (Sigma-Aldrich R2523) with a reaction total volume of 20 µl. Each reaction comprised 10 µl RedTaq ReadyMix PCR Reaction Mix, 1 µl (10 mM) of each forward and reverse primers, 50 ng of plant genomic DNA, made up to a total volume of 20 µl by sterile laboratory grade water. The specific forward (F) and reverse (R) PCR primers used are listed in Table 1.

**Table 1:** List of PCR primers used in the study

Gene/PCR	Primer left	Primer right	Description
nptII	GAG GCT ATT CGG CTA TGA CTG G	ATC GGG AGC GGC GAT ACC GTA	*NPT*II gene-specific primers to identify transgenic material
VirD2	TCA AGT AAT CAT TCG CAT TGT GCC	GCC GTG ACG AAG TGA AAT CTC	*VirD2* gene-specific primers to identify *Agrobacterium*
Pv actin	GTG ATA ATG GGA CCG GAA TG	TGC TTC CGT CAA CAA AAC AG	*Primula vulgaris*-Actin used to assess genomic DNA quality

The *NptII* PCR was used to identify transgenic plant material. The reaction conditions were as follows: denaturation at 94 °C for 5 min, then 30 cycles of 94 °C, 30 s denaturation; 60 °C for 30 s annealing and 72 °C for 1 min 15 s extension and a final extension at 72 °C for 5 min. The expected amplicon size for *NptII* PCR was 700 bp.

PCR analysis for the *Agrobacterium tumefaciens* Ti *VirD2* gene was performed on all plant genomic DNA extractions to check that there was no remaining *Agrobacterium* contamination. The *VirD2* PCR conditions were as follows: denaturation at 94 °C for 5 min, then 30 cycles of 30 s at 94 °C; 30 s annealing at 55 °C and 1 min extension at 72 °C, and a final extension at 72 °C for 5 min. The expected VirD2 amplicon size was 487 bp.

The quality of the genomic DNA used for this study was tested using primers specific for the endogenous *P. vulgaris Actin* gene. The *Pv Actin* reaction conditions were as follows: denaturation at 95 °C for 3 min, then 35 cycles of 30 s at 95 °C, 30 s annealing at 55 °C and 30 s extension at 72 °C, and a final extension at 72 °C for 5 min. The expected amplicon for *Pv Actin* PCR was 500 bp.

Positive, negative and water controls were included in all PCR analyses. The amplified products were resolved on a 1% agarose gel containing ethidium bromide at 0.1 μg ml^−1^ in 0.5 × TBE buffer.

#### Southern blot analysis

10 µg DNA was digested with 30 units of *Hind*III or *Eco* RI (both of which cut once in the T-DNA) with 3 µL 10X buffer in a total volume of 30 µL at 37 °C (Invitrogen/Life Technologies) overnight ~ 16 h. The digested DNA was separated on a 0.8% agarose gel at 3 V/cm overnight. An alkaline Southern blot was assembled [52] and the DNA transferred onto a Hybond-N + membrane (Amersham) by capillary transfer according to the manufacturer’s instructions. Previously-PCR amplified, ^32^P randomly labelled probe from the coding region of the *NptII* marker gene (700 bp) was used for hybridisation. The probe had been amplified using the primers nptII_F and nptII_R (Table 1) for *NptII* (see above section PCR). Labelling was carried out using a Ready-To-Go DNA labelling Beads (-dCTP) (GE Healthcare, Product code: 27-9240-01). Pre-hybridisation and hybridisation were carried out at 42 °C overnight in hybridisation buffer containing 50% formamide, 5× SSC, 5× Denhardt solution and 0.5% SDS. The membrane blot was then washed three times in a solution containing 2× SSC and 0.1% SDS at 42 °C. After the final wash, the membrane was sealed in a plastic bag and Kodak Biomax MR film was exposed to it for 4 days at room temperature 24 ± 1 °C. The film was developed using a medical film processor (SRX-101A, Konica Minolata Inc., Japan) and the image scanned and digitalised.

#### Analysis of transgene inheritance

Five T_0_ plants were out crossed to produce seed. Under aseptic conditions, these seed were surface sterilised as previously described and germinated on MS medium supplemented with 125 mg L^−1^ kanamycin within 100 mL Sterilin jars (~ 10 per jar). Seed germination took place in a controlled culture room at 21 ± 1 °C under cool white fluorescent light (50 μmol m^−2^ s^−1^) with a 16-h light period per day. Further analysis of transgene inheritance was obtained by crossing a T_0_ transgenic plant to produce seed and progeny grown to produce T_1_ plants. Leaves were collected from four T_1_ progeny, and genomic DNA was extracted. Transgene inheritance (*NptII* gene) was assessed by performing PCR analysis using the *NptII* gene specific primers and Southern analysis as described in the above section.
